# TIMP1 promotes microglia M2 polarization through MAPK pathway to ameliorate early brain injury after ischemia

**DOI:** 10.1186/s41065-025-00491-8

**Published:** 2025-07-02

**Authors:** Kangkang Zhao, Zizhao Huang

**Affiliations:** Department of Neurosurgery, Yulin Hospital, The First Hospital Affiliated to Xi’an Jiaotong University, Yulin, 719000 Shaanxi China

**Keywords:** Ischemic stroke, Neuroinflammation, TIMP1, MAPK

## Abstract

**Objective:**

To explore the molecular regulatory mechanisms and biomarkers in regulating early brain injury (EBI) and inflammatory response after ischemic stroke (IS).

**Methods:**

Gene expression profiles of GSE148350, GSE35338, and GSE58294 were analyzed to screen the core genes involved in EBI after IS. Middle cerebral artery occlusion and reperfusion (MCAO/R) model and oxygen and glucose deprivation and reoxygenation (OGD/R) model were used to construct in vivo and in vitro IS models. In MCAO/R model, the effects of tissue inhibitor of metalloproteinase-1 (TIMP1) were investigated by Zea longa score, brain water content assessment and histological analysis. In OGD/R model, after TIMP1 was overexpressed in BV2 cells, M1 and M2 polarization markers (iNOS and Arg-1) in BV2 cells were detected by Western blot, and the effects of BV2 on the viability and apoptosis of HT22 cells were evaluated by cell counting kit-8 and flow cytometry, respectively. Additionally, the effects of TIMP1 overexpression on MAPK pathway in BV2 cells were also detected by Western blot.

**Results:**

Two core genes, TIMP1 and vimentin (VIM) were screened from 254 differentially expressed genes in IS. TIMP1 was closely associated with the dysregulation of immune cell infiltration. TIMP1 overexpression significantly mitigated MCAO/R-induced neurological dysfunction, brain edema, neuronal apoptosis and inflammatory response in rats. In vitro, it was revealed that TIMP1 overexpression in BV2 cells increased viability and inhibited apoptosis of HT22 cells. In BV2 cells, TIMP1 overexpression promoted the expression of Agr-1 and inhibited the expression of iNOS. In addition, overexpression of TIMP1 inhibited OGD/R-induced increases in the phosphorylation levels of p38, JNK and ERK proteins in BV2 cells.

**Conclusion:**

This study identified a post-IS EBI regulator, TIMP1. TIMP1 promotes M2 polarization of microglia and ameliorate neurological injury after IS by inactivating MAPK signaling pathway.

**Supplementary Information:**

The online version contains supplementary material available at 10.1186/s41065-025-00491-8.

## Introduction

Stroke is one of the leading causes of death and disability worldwide. The World Health Organization estimates that about 150,000 new cases suffer from stroke each year, and 70% of those die or become permanently disabled [1, 2]. Ischemic stroke (IS) accounts for more than 80% of stroke cases [2]. IS is mainly caused by narrowing or blocking of arteries in the brain, leading to local ischemia and hypoxia, causing an inflammatory response and irreversible neuronal death. At present, the main treatment for IS is still thrombolysis or vascular interventional therapy [3], but due to the narrow therapeutic window and high risk, the therapeutic effect is still unsatisfactory. Therefore, there is an urgent need to identify new therapeutic targets to improve the clinical outcomes in patients with IS.

The pathophysiology of early brain injury (EBI) after IS is very complex, including calcium overload, excitatory amino acid neurotoxicity, oxidative stress and inflammatory response [4–7]. When ischemia occurs, microglia/macrophages are activated and recruited to the lesion site, presenting two phenotypes: “classically activated” M1 type and “alternatively activated” M2 type [8, 9]. M1 cells express and release pro-inflammatory mediators, which aggravate the inflammatory injury of neurons, and reduce the integrity of the blood-brain barrier (BBB); M2 cells secrete anti-inflammatory mediators that promote tissue repair [8–10]. Thus, modulating the immune microenvironment by shifting microglial activation from M1 to M2 in the peri-infarction area during the early stages of ischemic injury may serve as a promising therapeutic strategy for ameliorating EBI after IS.

In this study, we aimed to identify novel therapeutic targets for EBI post-IS. IS-related datasets from the Gene Expression Omnibus (GEO) database were analyzed, and core genes were screened from differentially expressed genes (DEGs). Additionally, the biological functions of the core target, tissue inhibitor of metalloproteinase-1 (TIMP1), were investigated through in vitro and in vivo experiments.

## Materials and methods

### Data collection and processing

Six stroke-related datasets GSE148350, GSE35338, GSE58294, GSE190171, GSE30655 and GSE107983 were downloaded from the GEO database. GSE148350 (platform: GPL6247) included sham and IS samples of 3-month old rats and 12-month old rats, with 3 samples each. In this study, only microglia samples separated from the same hemisphere 3 days after IS were included. This dataset was used to identify DEGs in microglia during EBI after IS. GSE35338 (platforms: GPL1261), GSE58294 (platform: GPL570), GSE190171 (platform: GPL24247), GSE30655 (platforms: GPL1261) and GSE107983 (platform: GPL6246) served as the external validation datasets. In GSE35338 dataset, the mice were injected intraperitoneally with 5 mg/kg lipopolysaccharide (LPS) to induce neuroinflammation (normal saline was used for control), and astrocytes were purified for gene expression profiling; additionally, the mice received received middle cerebral artery occlusion (MCAO) or sham surgery, and astrocytes were purified for gene expression profiling. GSE58294 dataset included blood samples from 23 controls and 69 patients with cardioembolic stroke. The dataset was used for core gene expression validation, gene set enrichment analysis (GSEA), and immunoinfiltration analysis. GSE190171 dataset included microglia samples from 5 Sham and 4 MCAO mice. GSE30655 dataset included brain tissue samples from 3 Sham and 7 MCAO mice. GSE107983 dataset included microglia samples from 4 mice with middle cerebral artery occlusion/reperfusion (MCAO/R) and 4 mice in the Sham group.

### Analysis and functional enrichment analysis of DEGs

Using sangerbox platform (http://vip.sangerbox.com/home.html) [11], DEGs in GSE148350 were analyzed. The threshold was set to “|log_2_fold change(FC)| > 1, *P* < 0.05”. DAVID database (https://david.ncifcrf.gov/summary.jsp) was used for Gene Ontology (GO), Kyoto Encyclopedia of Genes and Genomes (KEGG) and Reactome enrichment analysis. GO included three items, namely biological processes (BP), molecular functions (MF), and cellular components (CC). *P* < 0.05 was considered to be a significant difference level in enrichment results.

### Protein-protein interaction (PPI) network construction and visualization

PPI network of the DEGs was constructed using the STRING database (https://cn.string-db.org/). The.txt file was then imported into Cytoscope software to calculate the topological indicators including degree centrality (DC), closeness centrality (CC) and between centrality (BC) using cytoNAC plug-in. The PPI network was visualized after adjusting node color, font size, and node size according to DC, BC, and CC. In this study, the median values of DC, BC and CC were used as thresholds, and 15 core genes were obtained through two rounds of screening.

### GSEA

GSEA was carried out using sangerbox platform [11]. The samples were divided into high expression group and low expression group according to the expression level of genes. With “c2.cp.kegg.v7.4.symbols.gmt” and “c7.immunesigdb.v7.4.symbols.gmt” as the reference gene sets, the relevant pathways and biological processes were evaluated. *P* < 0.05 and false discovery rate (FDR) < 0.25 were considered statistically significant.

### Immune cell infiltration analysis

Using BioBean platform (http://sxdyc.com/immuneSsgseaScore), single sample GSEA (ssGSEA) algorithm was used for scoring 28 kinds of immune cells based on the characteristic genes [12]. Then, the results of immune cell infiltration analysis and pearson correlation coefficient between immune cell score and the genes were analyzed and visualized on sangerbox platform.

### Animal and MCAO/R model construction

Male Sprague-Dawley rats (280–320 g, Hibio, Hangzhou, China) were fed in a standardized condition: had free access to food and water, 25–28 °C, 12 h/12 h light/dark cycle, relative humidity 50–60%. The animal experiment protocol for this study was approved by the Yulin Hospital, First Hospital Affiliated to Xi’an Jiaotong University (Approval No. YLYY-2024007).

In experiment 1, the rats were divided into two groups: sham group (5 rats) and MCAO/R groups (9 rats; 3 rats per group). The brain tissues of the rats in MCAO/R groups were collected at 24 h (3 rats), 48 h (3 rats) and 72 h (3 rats) after modeling, respectively, and the expression of TIMP1 was detected by Western blot.

In experiment 2, the rats were divided into 4 groups: control group (sham group, 12 rats), model group (MCAO/R group, 12 rats), model + negative control group (MCAO/R + NC, 12 rats) and model + TIMP1 overexpression group (MCAO/R + TIMP1, 12 rats). For the MCAO/R + NC group and MCAO/R + TIMP1 group, 10 µL AAV-NC (6.5 × 10^9^ viral particles /mL) and AAV-TIMP1 (6.5 × 10^9^ viral particles /mL) were injected into the ipsilateral lateral ventricle, respectively, 30 days prior to modeling. The MCAO/R rat model was constructed according to Longa’s method [13]. The middle cerebral artery was not blocked in the sham group. Then the neurological injury, cerebral edema, histological change and gene expression change of the brain tissues of the rats in these groups were compared.

### Zea-Longa score

The modified Zea-Longa scoring system was used to evaluate the neurobehavioral functions of the rats [13]. The Zea-Longa score was measured 24 h after MCAO/R. The scoring criteria were as follows: 0: no obvious defects; 1 score: forelimb flexion, mild nerve damage; 2 points: one-way hover, moderate nerve damage; 3 points: fall to the hemiplegic side, neurological function was seriously damaged; 4 points: inability to walk autonomously or lack of awareness.

### Brain water content assessment

Cerebral edema was assessed by using the “wet and dry” method. In short, after the rats were killed, the brains were removed and quickly weighed to obtain the wet weight. After drying at 105 °C for 24 h, and the tissues were weighed to determine dry weight. The brain water content was calculated as follows: [(wet weight - dry weight)/ wet weight] ×100%.

### Hematoxylin-Eosin (HE) staining

After 24 h of modeling, the rats were killed and the brain tissues were collected. The brain tissue was fixed with 4% paraformaldehyde, embedded in paraffin, and cut into Sect. (5 μm thickness). After dewaxing, HE staining was performed, and histopathological changes of the brain tissues of each group were observed under a 200x optical microscope (Olympus).

### Western blot

Protein was extracted from homogenized brain tissues or cells using RIPA lysis buffer (Beyotime). The protein was quantified by a BCA protein quantification kit (Solarbio). After the protein samples were adjusted to the same concentration, the same amount of protein (30 µg per lane) was separated by electrophoresis and then transferred to polyvinylidene fluoride (PVDF) membranes (Millipore). After blocking, the membranes were incubated with antibodies. The primary antibodies: anti-TIMP1 (ab179580, 1: 1000, Abcam), anti-GAPDH (ab9485, 1:1000, Abcam). anti-Bcl-2 (ab182858, 1:1000, Abcam), anti-Bax (ab32503, 1:1000, Abcam), anti-iNOS (ab178945, 1:1000, Abcam), anti-Agr-1(#93668, 1:1000, Cell Signaling Technology), anti-p-p38(ab4822, 1:1000, Abcam), anti-p38 (ab170099, 1:1000, Abcam), anti-p-JNK (#4668, 1:1000, Cell Signaling Technology), anti-JNK (#9252, 1:1000, Cell Signaling Technology), anti-p-ERK (ab131438, 1:1000, Abcam), anti-ERK (ab32537, 1:1000, Abcam). The secondary antibody was Goat Anti-Rabbit IgG H&L (HRP) (ab205718, 1:2000, Abcam, Shanghai, China). ECL Western Blotting Substrate (Solarbio) was used for the development of the protein bands. Finally, the imaging system (Bio-Rad, Hercules, CA, USA) was used for imaging and photographing, and the bands were quantified using Image J software.

### Quantitative RT-PCR (qRT-PCR)

Total RNA was extracted from brain tissue or cells with TRIzol reagent (Takara). RNA was reverse-transcribed into cDNA using a PrimeScript™ RT reagent Kit (Takara). qRT-PCR was performed using TB Green™ Premix Ex Tag™ II kit (Takara) on ABI 7500 real-time system (Applied Biosystems). The relative expression of target genes was normalized to endogenous control GAPDH expression and quantified by the 2^−△△ct^ method. Primer sequences were as follows: TNF-α-forward: 5 ‘-ATGGCCTCCCTCTCATCAGT-3’, reverse: 5’-TGGTTTGCTACGACGTGGG-3’; IL-6-forward: 5’- CGTGGAAATGAGAAAAGAGTTGTGC-3’, reverse: 5’- GGTACTCCAGAAGACCAGAGGA-3’; IL-10-forward: 5’-AGGGTTACTTGGGTTGCC-3’, reverse: 5’-GGGTCTTCAGCTTCTCTCC-3’; GAPDH-forward: 5’-TGTGTCCGTCGTGGATCTGA-3’, reverse: 5’-TTGCTGTTGAAGTCGCAGGAG-3’.

### Cell culture and oxygen and glucose deprivation (OGD) model

Mouse microglia BV2 cell line and hippocampal neuron HT22 cell line (National Collection of Authenticated Cell Cultures, Shanghai, China) were cultured in Dulbecco’s modified Eagle’s medium (DMEM, Gibco) supplemented with high glucose, 10% fetal bovine serum (FBS, Thermo Fisher Scientific), 100U/mL penicillin and 0.1 mg/mL streptomycin (Beyotime) at 37 °C in 5% CO_2_. BV-2 microglial cells were infected with AAV-NC or AAV-TIMP1 (both at 6.5 × 10^9^ virus particles/mL; Vector Biolabs) at a concentration of 150 µL/well. When the multiplicities of infection (MOI) were 100, the infection rate of cells reached over 80% [14, 15]. The transfected cells were incubated for an additional 24 h prior to OGD. For OGD induction, BV2 cells were cultured in a glucose-free medium and then transferred to a sealed anoxic chamber containing a mixture of 95% N_2_, 5% CO_2_, and 1%O_2_, and cultured at 37 °C for 4 h. The cells were then reoxygenated for 24 h incomplete medium before subsequent assays.

### Cell viability assay

A Cell Counting Kit-8 (CCK-8) (Beyotime) was applied to evaluate neuronal viability. In short, the cells were inoculated into a 96-well plate at a density of 1 × 10^6^ cells/well. After OGD, CCK-8 reagent was added to the medium (10 µL per well) and cultured at 37℃ for 2 h. Finally, the absorbance was measured at 450 nm wavelength with a microplate reader. Cell survival was shown as a percentage of the control group.

### Flow cytometry

Apoptosis of HT22 cells were evaluated with an Annexin V-FITC Apoptosis Assay Kit (Beyotime). After OGD, 100 µL HT22 cell suspension was added with 5 µL AnnexinV-FITC staining solution and 10 µL Annexinv-FITC staining solution (PI), respectively, and fully mixed. Cell apoptosis was measured on the FACS Calibur Flow Cytometer (BD Biosciences) immediately after incubation at 4℃ for 30 min without light and washing.

### Statistical analysis

All data of in vitro and in vivo assays were expressed as “mean ± standard deviation”. Statistical analysis was performed using GraphPad Prism software (version 8; GraphPad, La Jolla, CA, USA). One-way analysis of variance (ANOVA) followed by Tukey’s *post-hoc* test was performed for comparisons. *P* < 0.05 was considered statistically significant.

## Results

### Screening of DEGs after IS

**Fig. 1:**
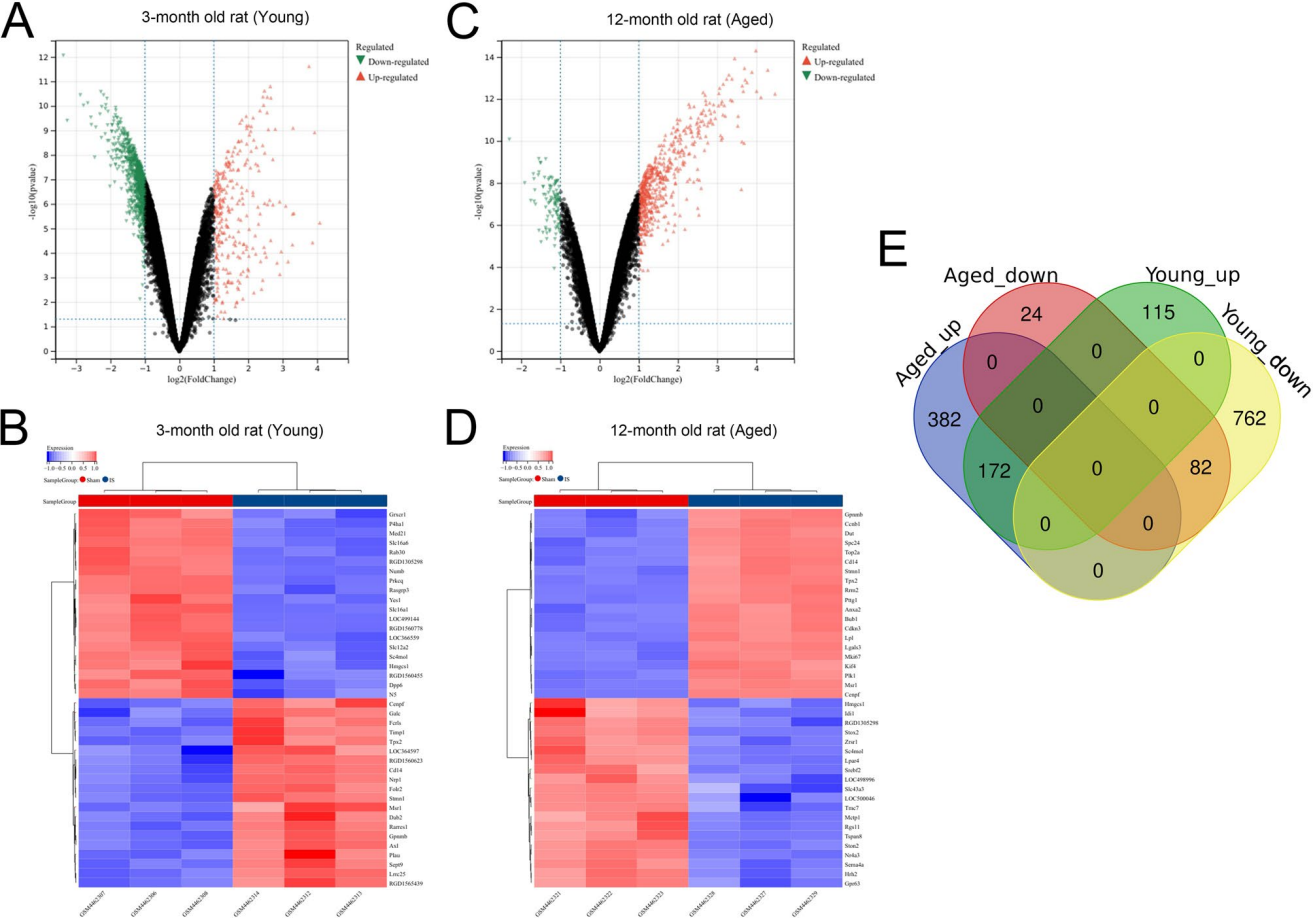
Screening the DEGs in microglia after IS. **A**. The volcanic map of the DEGs in the microglia of 3-month old (young) rat with IS and sham sample from GSE148350. Green dots represent down-regulated genes and red dots represent up-regulated genes. **B**. The expression profile of top 20 up-regulated genes and top 20 down-regulated genes in the microglia of young rats with IS. **C**. The volcanic map of the DEGs in the microglia of 12-month old (old) rat with IS and sham sample from GSE148350. Green dots represent down-regulated genes and red dots represent up-regulated genes. **D**. The expression profile of top 20 up-regulated genes and top 20 down-regulated genes in the microglia of old rats with IS. **E**. A Venn diagram was used to obtain the DEGs in both young and old rats

**Fig. 2:**
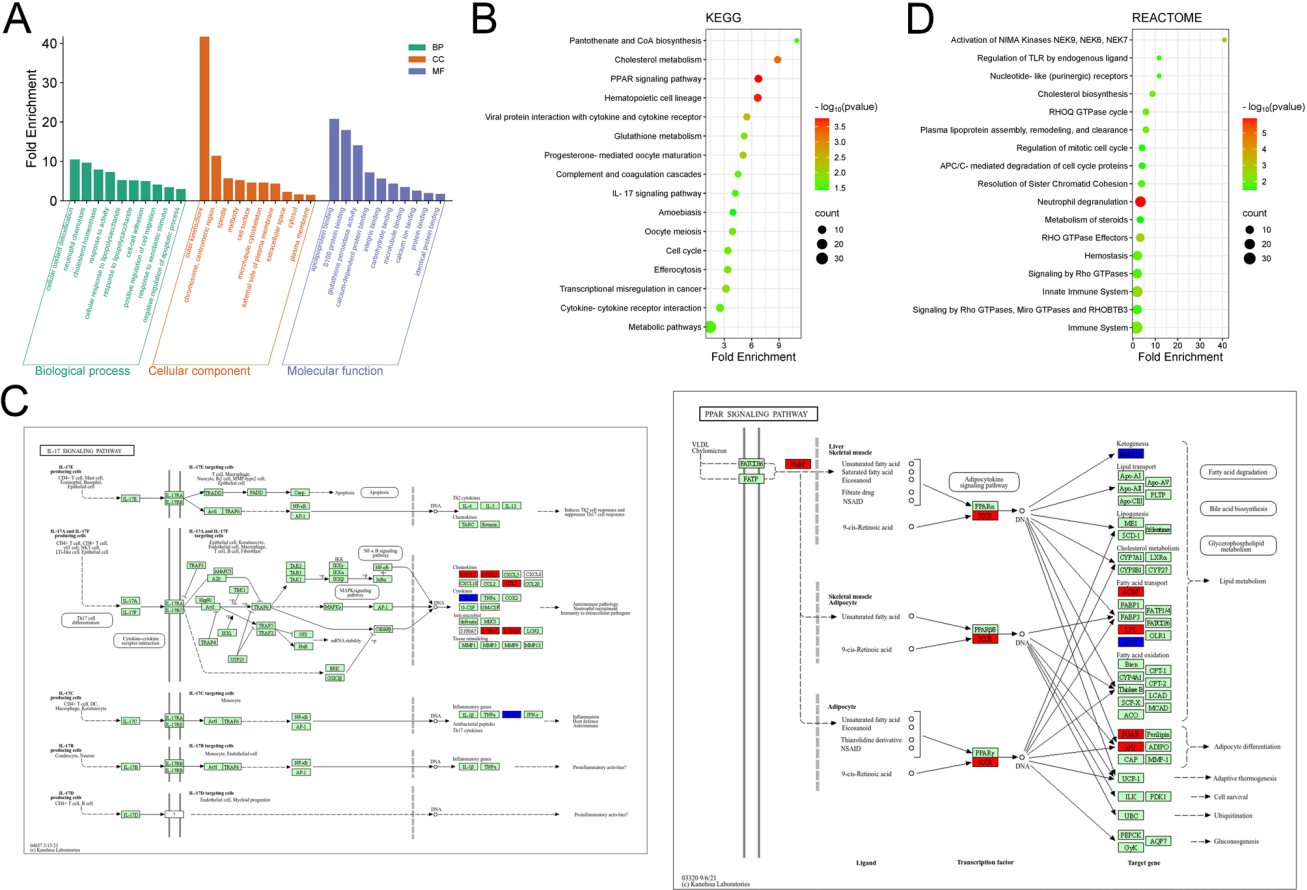
Functional enrichment analysis of the DEGs. **A**. The bar charts of the top 10 BP, CC and MF items from the GO analysis of the DEGs. **B**. The bubble map of the top 10 items from KEGG pathway enrichment analysis of the DEGs. **C**. IL-17 signaling pathway (rno04657) and PPAR signaling pathway (rno03320). The red marks significantly up-regulated genes in GSE148350, and the blue marks the significantly down-regulated genes The bubble map of the top 10 items from Reactome enrichment analysis of DEGs

Gene expression profiles of brain tissue samples, from the rats at 3 days after IS and the rats in sham group in dataset GSE148350, were analyzed. 287 up-regulated and 844 down-regulated genes were obtained from young rat brains (Fig. 1A-B). 554 up-regulated and 106 down-regulated genes were obtained from aged rat brains (Fig. 1C-D). A total of 172 up-regulated genes and 82 down-regulated genes were obtained in the intersections, totaling 254 DEGs (Fig. 1E). GO analysis showed that DEGs were associated with 131 BP, including cellular response to lipopolysaccharide (GO:0071222), cellular oxidant detoxification (GO:0098869), neutrophil chemotaxis (GO:0030593), apoptotic process (GO:0043066), cell migration (GO:0030335) and cell-cell adhesion (GO:0098609) (Fig. 2A); 40 CC, including external side of plasma membrane (GO:0009897), chromosome, centromeric region (GO:0000775) and extracellular space (GO:0005615) (Fig. 2A); 26 MF, including apolipoprotein binding (GO:0034185), S100 protein binding (GO:0044548) and glutathione peroxidase activity (GO:0004602) (Fig. 2A). 16 pathways were obtained by KEGG enrichment analysis, including hematopoietic cell lineage (rno04640), cholesterol metabolism (rno04979), PPAR signaling pathway (rno03320) and IL-17 signaling pathway (rno04657) (Fig. 2B-C). Reactome pathway enrichment analysis retrieved 17 items, including neutrophil degranulation (R-RNO-6798695), innate immune system (R-RNO-168249) and RHO GTPase Effectors (R-RNO-195258) (Fig. 2D). These results suggested that EBI of IS was associated with immune response, oxidative toxicity, cell polarization, and apoptosis.

### PPI construction and screening of core genes in EBI of IS

**Fig. 3:**
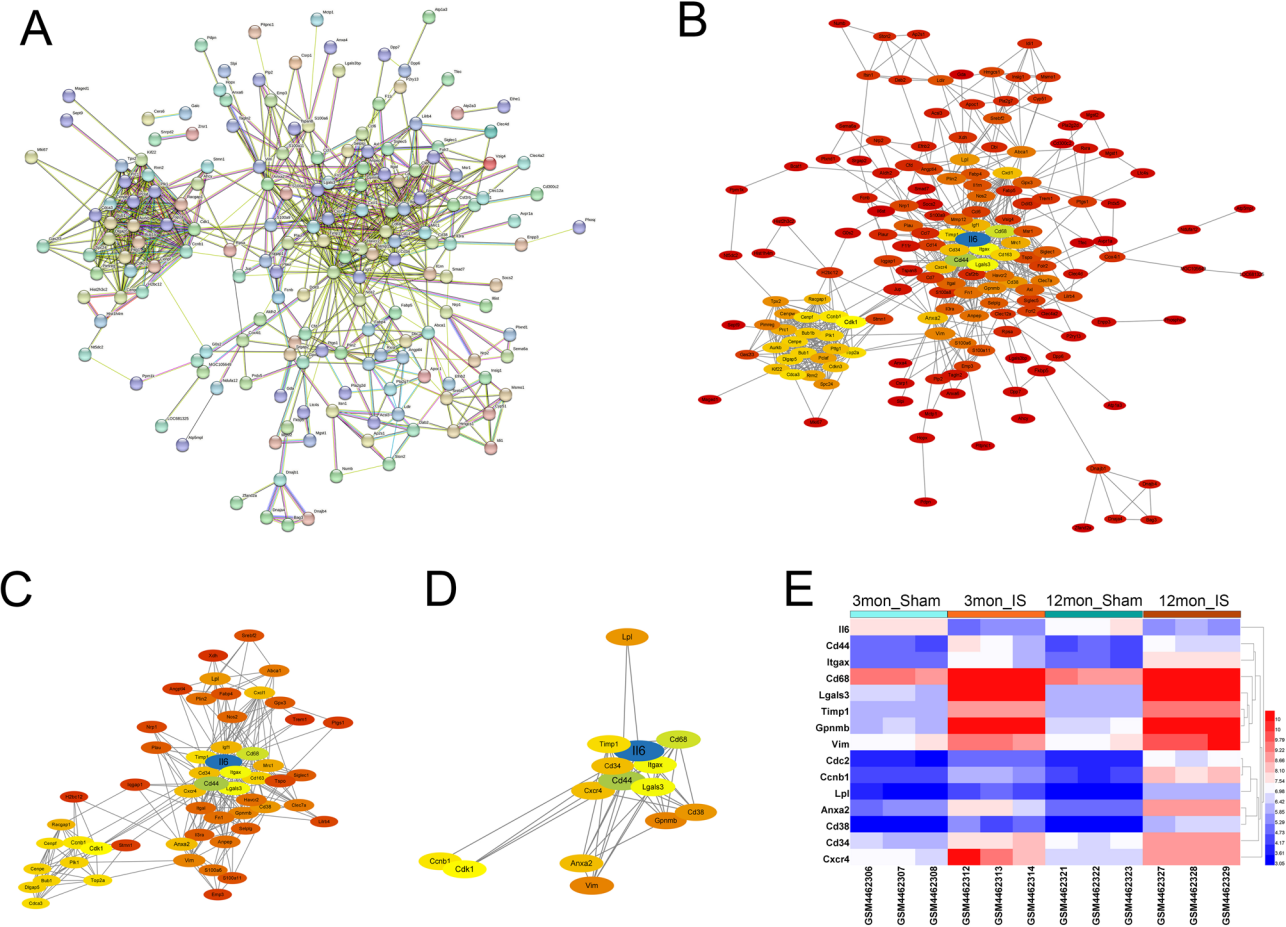
PPI construction and core gene screening. **A**. The PPI network of DEGs constructed using STRING database. **B**. Cytoscope software was used to visualize the PPI network. The depth of the node color deepens as the DC value increases. The node size increases as CC increases. The font size of the gene increases as the BC increases. **C**. The PPI network, obtained from the first screening, contains 55 nodes and 286 edges. **D**. The PPI network, obtained after the second screening, contains 15 nodes and 54 edges. **E**. The heat map shows the expression profiles of 15 core genes in GSE148350 dataset

**Fig. 4:**
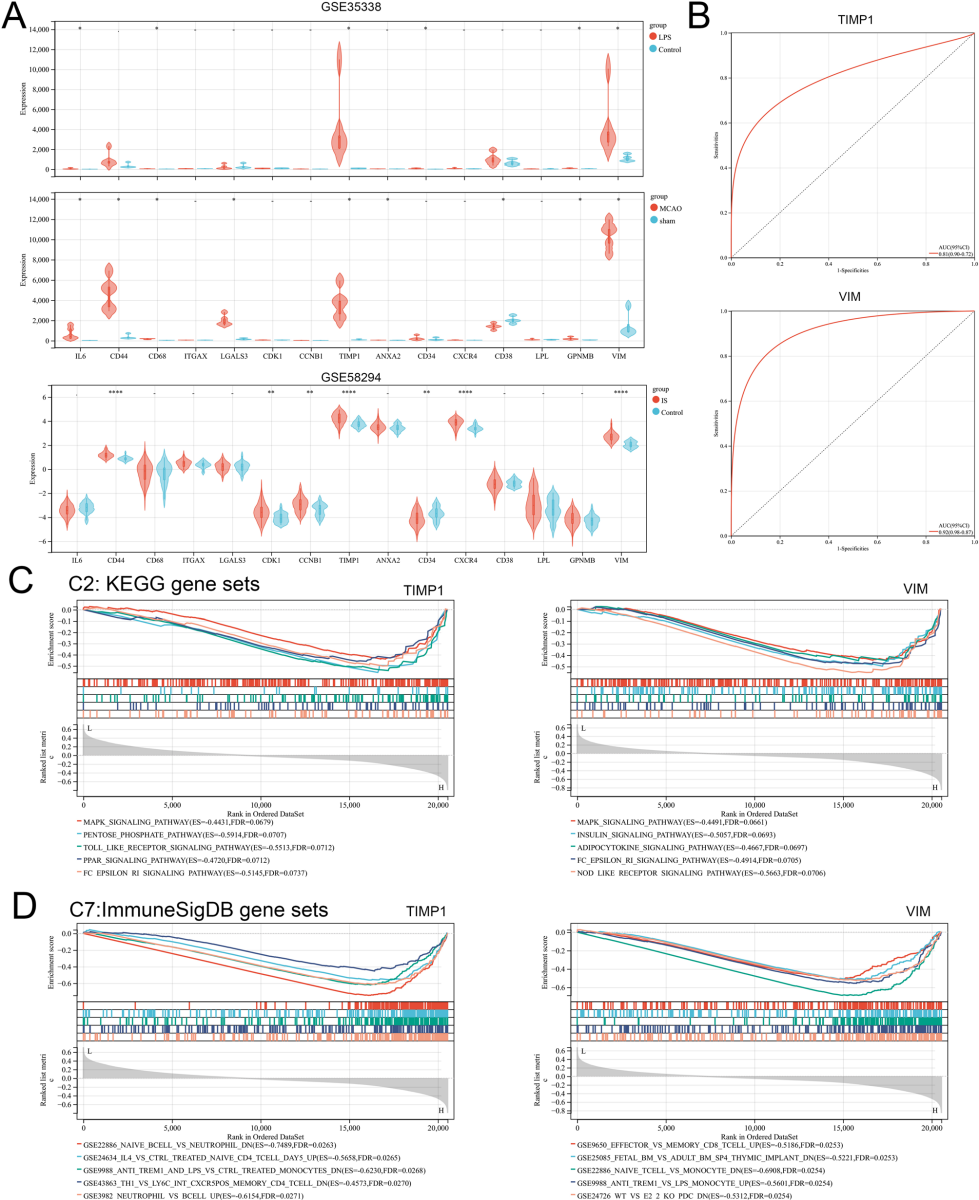
Verification of core genes in external datasets. **A**. Expression of 15 core genes in the external dataset GSE58294 and GSE35338 in IS and control groups. **B**. The diagnostic value of TIMP1 and VIM in IS was analyzed by ROC. **C**. The GSEA plots showed the top 5 KEGG pathways negatively correlated with TIMP1 and VIM expression. **D**. The GSEA plots showed the top 5 immune-related processes negatively correlated with TIMP1 and VIM expression

The PPI network of DEGs was constructed using STRING database, with 242 nodes and 737 edges (Fig. 3A). This was followed by further visualization and core gene screening via cytoscope software (Fig. 3B). The first round of screening was based on the median values of BC, CC, and DC (BC > 51.99, CC > 0.32, and DC > 6), resulting in a network of 55 nodes and 286 edges (Fig. 3C). The second round of screening was based on BC > 457.12, CC > 0.38, and DC > 13, and a network of 15 nodes and 54 edges was obtained (Fig. 3D). 15 genes (IL6, CD44, Cd68, ITGAXI, LGALS3, CDK1, CCNB1, TIMP1, ANXA2, CD34, CXCR4, CD38, LPL, GPNMB and VIM) were identified as the core genes of EBI after IS, and most of these core genes were highly expressed after IS in GSE148350 (Fig. 3E), and this was verified with multiple external datasets (Fig. 4A and Supplementary Fig. 1A-C). Furthermore, ROC analysis was performed using GSE58294, and the results showed that TIMP1 (AUC:0.92, 95%CI:0.98 − 0.87) and VIM (AUC:0.81, 95%CI:0.90 − 0.72) were with good diagnostic value for IS (Fig. 4B). Then GSEA was performed on TIMP1 and VIM. For C2: KEGG gene set, it showed that TIMP1 and VIM were closely related to mitogen-activated protein kinase (MAPK) signaling pathway (Fig. 4C). For C7 immune-related gene set, it showed that TIMP1 was related to neutrophils, T cells and B cells, and VIM was associated with T cell response (Fig. 4D).

### Immune cell infiltration analysis

**Fig. 5:**
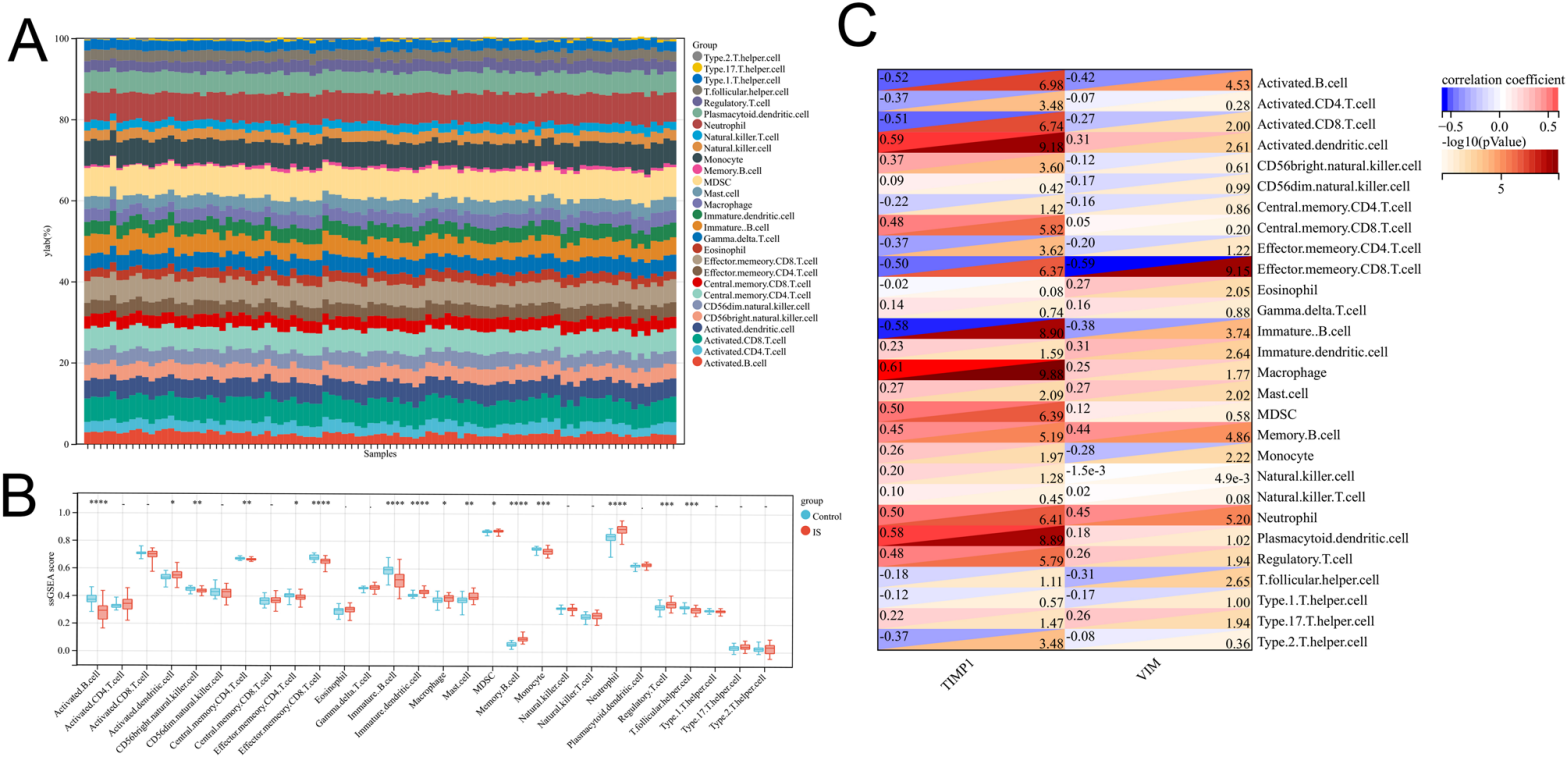
Analysis of immune cell infiltration. **A**. Heatmap of the 28 immune cell proportions in each sample. **B**. Box plot of the infiltration of 28 immune cells in IS and control groups. **C**. The heat map showed the correlation between the infiltration of 28 immune cells and the expression levels of TIMP1 and VIM

ssGSEA algorithm was further used for immune infiltration analysis of the GSE58294 dataset (Fig. 5A), and there were significant differences between the IS and the control in 16 kinds of immune cells. Activated dendritic cell, immature dendritic cell, mast cell, myeloid-derived suppressor cell (MDSC), neutrophil, and regulatory T cells in IS group was significantly higher than that in control (Fig. 5B). Correlation analysis found that TIMP1 was significantly negatively correlated with activated B cell, activated CD8 T cell, effector memeory CD8 T cell, and immature B cell (*r*≤-0.5, *P* < 0.05), and it was significantly positively correlated with activated dendritic cell, macrophage, MDSC, neutrophil and plasmacytoid dendritic cell (*r* ≥ 0.5, *P* < 0.05) (Fig. 5C). VIM was significantly negatively correlated with effector memeory CD8 T cell (*r*=-0.59, *P* = 1.0e-9.15) (Fig. 5C). These results suggested that TIMP1 played a more important role than VIM, in regulating the immune microenvironment and inflammatory response in EBI of IS.

### Overexpression of TIMP1 improves neurological function, ameliorates brain injury and inflammatory response in MCAO/R rats

**Fig. 6:**
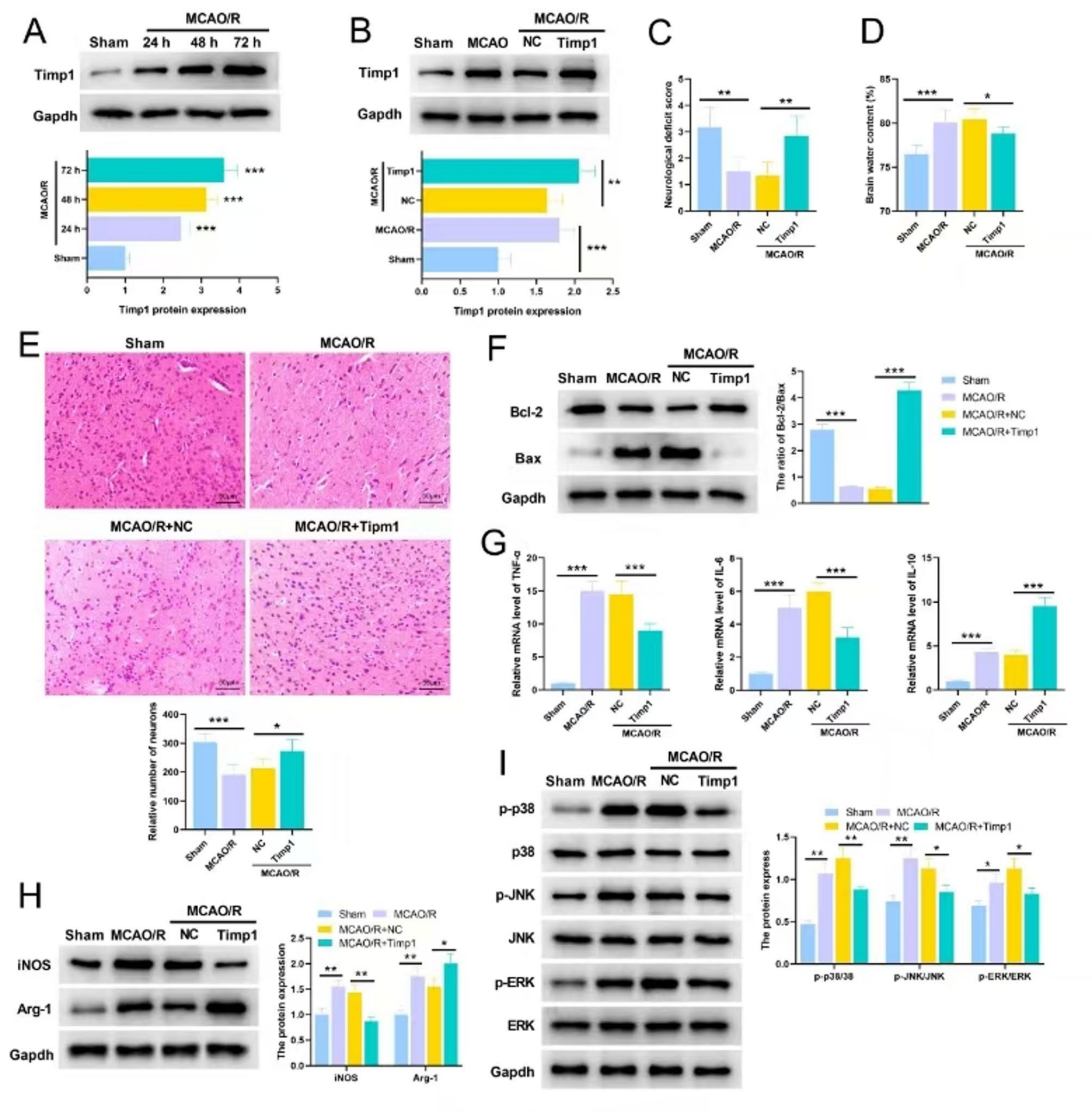
Overexpression of TIMP1 improves neurological function, and attenuates brain injury and inflammatory response in rats with ischemia stroke. **A-B**. Western blot assay to detect the protein expression level of TIMP1 in the brain tissues of the rats in different groups (3 rats in each group). **C**. Longa score was used to evaluate neurological deficits in rats (6 rats in each group). **D**. Wet and dry method was used to evaluate cerebral edema in rats (6 rats in each group). **E**. HE staining was used to evaluate neuronal injury in rats (6 rats in each group). **F**. Western blot assay to detect the expression levels of apoptosis-related proteins Bcl-2 and Bax in rat brain tissues (6 rats in each group). **G**. mRNA expression levels of TNF-α, IL-6 and IL-10 in rat brain were detected by qRT-PCR (6 rats in each group). **H**. Western blot assay was performed to detect the expression of iNOS and Arg-1, markers of M1 and M2 polarization in brain tissue (6 rats in each group). **I**. Western blot assay was performed to detect the phosphorylation levels of key proteins in MAPK pathway, including p38, JNK and ERK in brain tissue (6 rats in each group) *P < 0.05, **P < 0.01, ***P < 0.001

At 24 h, 48 h and 72 h after the IS rat model was constructed by MCAO/R, the expression of TIMP1 was gradually increased (Fig. 6A). Previous studies have shown that high expression of TIMP1 can inhibit inflammatory response, alleviate BBB injury, and repress EBI induced in subarachnoid hemorrhage model [16, 17]. In this study, rats with high expression of TIMP1 were constructed, and then MCAO/R models were established. In the rats with TIMP1 overexpression, the level of TIMP1 was significantly increased (Fig. 6B). The neurological function score of rats with TIMP1 overexpression was significantly higher than that of the MCAO/R + NC group (Fig. 6C), and the brain water content in TIMP1 overexpression group was slightly lower than that of the MCAO/R + NC group (Fig. 6D). HE staining showed that compared with the NC group, the number of neurons in the cerebral cortex of rats with TIMP1 overexpression was significantly increased (Fig. 6E). Western blot showed that TIMP1 overexpression significantly increased the ratio of Bcl-2/Bax compared with the NC group (Fig. 6F). In addition, overexpression of TIMP1 significantly inhibited the mRNA expression of TNF-α and IL-6, and promoted the mRNA expression of IL-10 (Fig. 6G). These results suggest that overexpression of TIMP1 could repress IS-induced brain injury and neuroinflammation. The protein expression levels of iNOS and Arg-1 in the brain tissues were significantly increased after MCAO/R, and overexpression of TIMP1 significantly inhibited the expression of iNOS and promoted the protein expression of Arg-1 (Fig. 6H), suggesting TIMP1 promoted M2 polarization of microglia in the brain tissues of the rats. Additionally, the expression levels of p-p38, p-JNK and p-ERK in MCAO/R group were significantly higher than those of the sham group; the phosphorylation levels of p38, JNK and ERK in TIMP1 overexpression group were lower than those in the MCAO/R + NC group (Fig. 6I), implying up-regulation of TIMP1 inactivated MAPK pathway.

### Overexpression of TIMP1 can inhibit OGD-induced apoptosis of TH22 cells

**Fig. 7:**
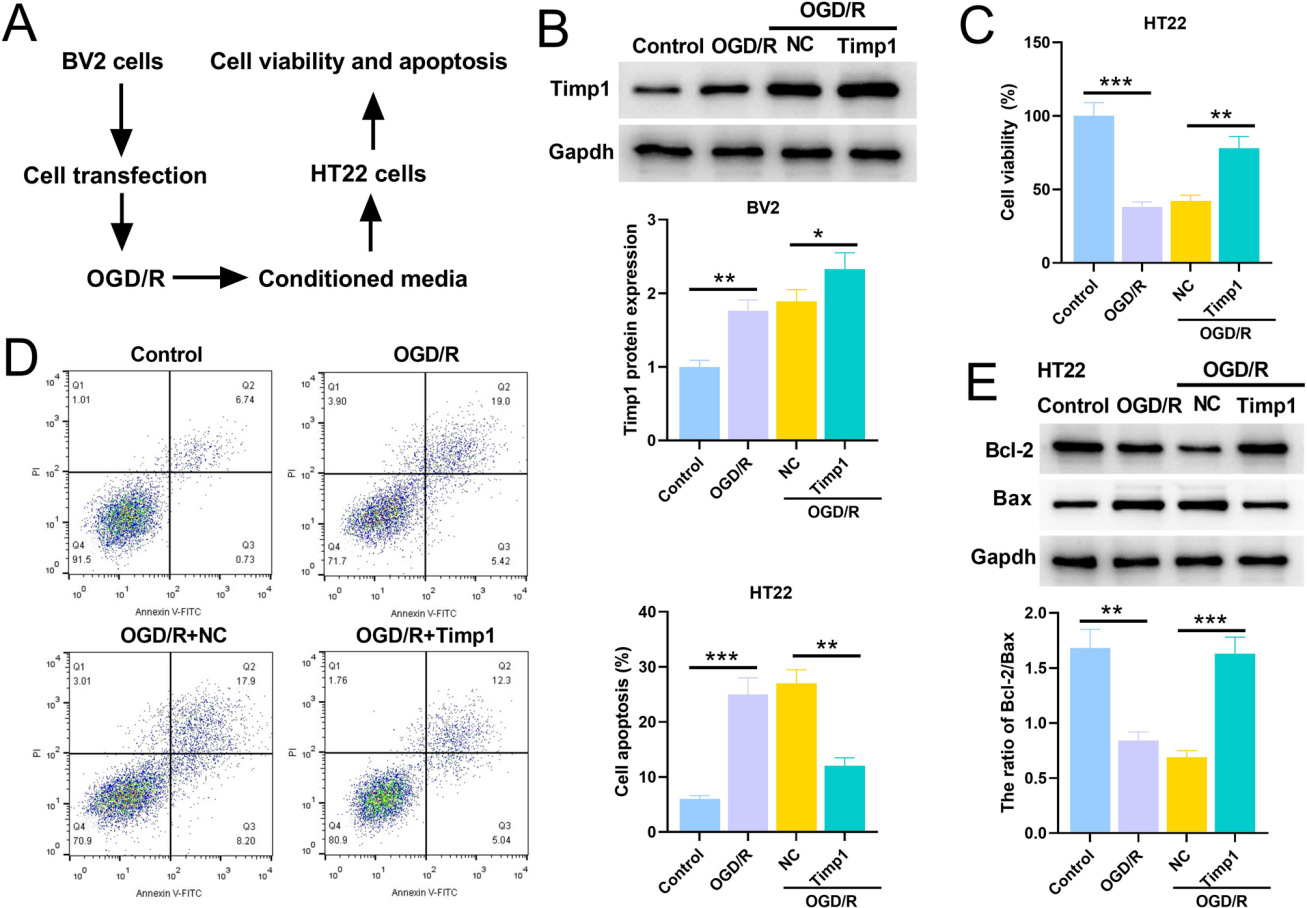
Overexpression of TIMP1 inhibits OGD-induced apoptosis of HT22 cells. **A**. The co-culture system of BV2 and HT22 cells in vitro. **B**. Western blot assay was used to detect the expression level of TIMP1 protein in BV2 cells. **C**. CCK-8 assay was used to detect the viability of HT22 cells in different groups. **D**. The apoptosis of HT22 cells in different groups was detected by flow cytometry. **E**. Western blot assay to detect the expression levels of apoptosis-related proteins Bcl-2 and Bax in HT22 cells of different treatment groups in HT22 cells **P < 0.01, ***P < 0.001

The neuroprotective function of TIMP1 was further explored through co-culture system of BV2 and HT22 cells (Fig. 7A). The TIMP1 overexpression vector was transfected into BV2 cells, and compared with the NC group, the expression of TIMP1 in the TIMP1 overexpression group was significantly increased (Fig. 7B). Compared with the control group, OGD significantly inhibited the viability of HT22 cells, while the viability of cells in the TIMP1 overexpression group was significantly increased compared with that in the NC group (Fig. 7C). Flow cytometry showed that overexpression of TIMP1 significantly reduced OGD-induced apoptosis of HT22 cells (Fig. 7D). Western blot showed that the ratio of Bcl-2/Bax after OGD treatment was significantly lower than that in the control group, while the ratio of Bcl-2/Bax after TIMP1 overexpression was significantly higher in HT22 cells (Fig. 7E).

### TIMP1 promotes M2 transformation of microglia by inhibiting the activation of MAPK signaling pathway

**Fig. 8:**
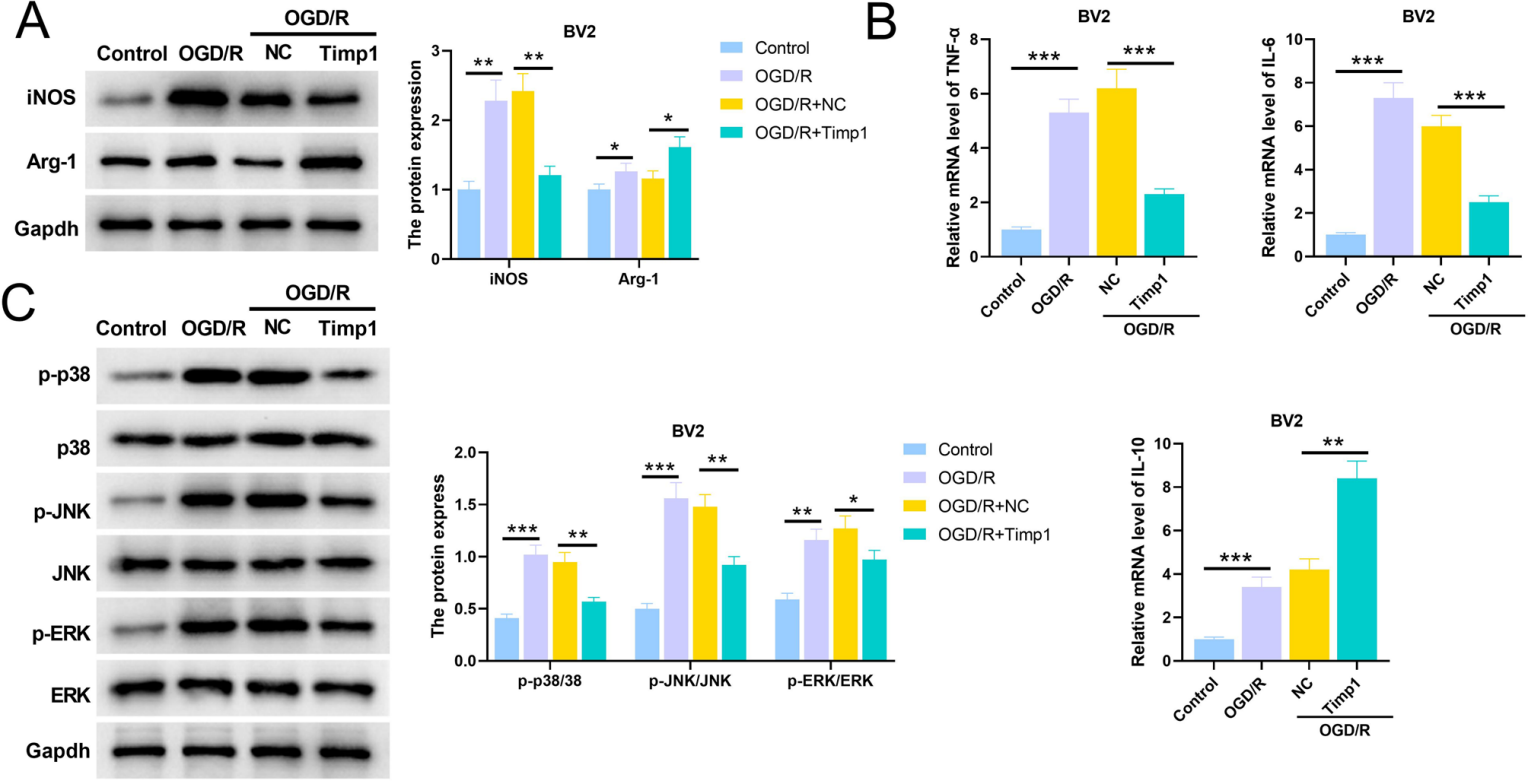
TIMP1 promotes M2 polarization of microglia by inhibiting the activation of MAPK pathway. **A**. Western blot assay was performed to detect the expression of iNOS and Arg-1, markers of M1 and M2 polarization in BV2 microglia. **B**. The mRNA expression levels of TNF-α, IL-6 and IL-10 in BV2 cells were detected by qRT-PCR. **C**. The phosphorylation levels of key proteins in MAPK pathway, including p38, JNK and ERK were detected by Western blot assay *P < 0.05, **P < 0.01, ***P < 0.001

The protein expression levels of iNOS and Arg-1 in BV2 cells were significantly increased after OGD, and overexpression of TIMP1 significantly inhibited the expression of iNOS and promoted the protein expression of Arg-1 (Fig. 8A). In BV2 cells, overexpression of TIMP1 significantly inhibited the OGD-induced increase of TNF-α and IL-6 mRNA expression, and promoted the mRNA expression of IL-10 (Fig. 8B). Subsequently, Western blot was used to detect the expression of key proteins of MAPK signaling pathway, and the results showed that the levels of p-p38, p-JNK and p-ERK after OGD were significantly higher than those of the control group; the phosphorylation levels of p38, JNK and ERK in TIMP1 overexpression group were lower than those in the NC group (Fig. 8C). These results implied that TIMP1 could promote the transformation of microglia from M1 type to M2 type and inhibit the activation of MAPK signaling pathway.

## Discussion

Neuroinflammation plays a crucial role in the occurrence, development and recovery of IS, which greatly affects the disease severity and prognosis of IS patients [18, 19]. From GSE148350 dataset, a total of 254 DEGs were screened. These DEGs were involved in the regulation of immune response, cellular oxidative toxicity, cell polarization, apoptosis and other processes, and were closely related to PPAR pathway and IL-17 pathway. The peroxisome proliferators-activated receptors (PPAR) pathway is a key transcriptional regulatory pathway that plays an important role in lipid metabolism, inflammatory response, and cardiovascular diseases [20]. PPAR inhibits inflammatory processes, and reduces brain damage and improve motor and cognitive outcomes [21]. In addition, PPAR is closely related to microglial activation and phenotypic transformation [22, 23]. Interleukin-17 (IL-17) is a family of cytokines that includes six members from IL-17 A to IL-17 F, most of which are involved in pro-inflammatory processes. After the occurrence of IS, a large number of injury-related factors and inflammatory factors activate IL-7 A. Subsequently, IL-7 A recruits neutrophils to infiltrate the central nervous system by inducing the release of inflammatory factors, damaging the integrity of BBB and promoting the development of stroke [24–26].

With cytoNCA, 15 core genes in EBI of IS were identified, including IL6, CD44, Cd68, ITGAXI, LGALS3, CDK1, CCNB1, TIMP1, ANXA2, CD34, CXCR4, CD38, LPL, GPNMB and VIM. With the exception of IL6, other genes were significantly overexpressed in IS samples of GSE148350. In the datasets of this study, it was found that the expression of IL6 in the IS group was significantly higher than that in the control group in astrocytes (GSE35338), human blood (GSE58294), and mouse brain tissue samples (GSE30655), while the expression of IL6 in rat microglia (GSE148350) and mouse microglia (GSE190171 and GSE107983) in IS group was lower, or not changed, compared with that in the control group. IL6 plays a bidirectional role in IS: on one hand it promotes inflammatory injury as a pro-inflammatory factor in the acute phase, and on the other hand, IL-6 functions as a neurotrophic factor in the sub-acute and chronic phases, promoting M2 polarization of microglia, regulating the immunosuppressive microenvironment, and promoting neurogenesis, angiogenesis and neuronal differentiation [27, 28]. Two EBI biomarkers TIMP1 and VIM were further obtained based on the data of two external GEO datasets. These two genes have certain diagnostic value for IS, and are related to inflammation and immune processes after IS.

Vimentin (VIM) is a cytoskeletal intermediate filament protein involved in many biological processes such as signal transduction, adhesion, migration, apoptosis and differentiation [29]. Extracellular VIM can induce macrophages to release inflammatory cytokines, such as TNF-α and IL-6 [30]. High circulating VIM level is associated with an increased risk of IS [31]. In addition, VIM is related to astrocyte reactivity after IS, which is necessary for axon regeneration and neuronal network recombination [32–34]. TIMP1 is a blood biomarker of IS, which is highly expressed in the acute stage of IS, and its elevated level is closely related to the increased risk of severe disability and death after IS [35]. This study implies that TIMP1 may be a key regulator involved in the occurrence and development of acute post-stroke inflammation. A mouse experiment shows that TIMP1 regulates endothelial barrier integrity by interacting with CD63/integrin β1 complex and has a protective effect on BBB breakdown induced by traumatic brain injury [36]. In addition, TIMP1 is an endogenous inhibitor of matrix metalloproteinases (MMPs), which can cause the inactivation of most MMPs [37]. MMP9 is known to promote neuroinflammation and accelerate neuronal degeneration [38]. MMP9/TIMP1 imbalance is a key factor in the etiology of IS [39]. Previous studies have suggested that regulating MMP9/TIMP1 imbalance can effectively inhibit the activation of microglia and neuronal cell death in OGD/R models [40, 41]. In this study, the results of immune cell infiltration analysis showed that TIMP1 was associated with the dysregulation of immune cell infiltration. Considering immune cell infiltration plays an important role in the inflammatory response and secondary brain injury after IS [42], our data further demonstrate the critical role of TIMP1 in post-IS EBI.

Subsequently, by constructing a MCAO/R model in rat, it was verified that TIMP1 overexpression could significantly attenuate the neurological impairment, brain edema, neuronal apoptosis and neuroinflammation. The IS model was further constructed with a co-culture system of BV2 and HT22 cells, and it was found that TIMP1 overexpression promoted M2 polarization and pro-inflammatory factor production of microglia, significantly increased neuronal viability, and inhibited neuronal apoptosis. GSEA showed that high TIMP1 expression was negatively correlated with MAPK signaling pathway. The MAPK signaling pathway can transmit signals from the cell membrane to the nucleus in response to different stimuli, regulating proliferation, differentiation, inflammation, and apoptosis [43]. The increased expression of MAPK plays a crucial role in the inflammatory activation process during cerebral ischemia [44, 45]. Inhibiting the activation of MAPK signaling pathway has been reported to suppress M2 polarization of microglia and inflammatory response [46, 47]. Previous studies have confirmed that up-regulation of TIMP1 is related to inhibition of MAPK signaling pathway [48]. In addition, MAPK signaling pathway is involved in the regulation of MMPs/TIMP balance, and inactivation of MAPK signaling pathway is related to the reduction of the ratio of MMP1/TIMP1 and MMP1/TIMP2 [49]. As expected, in the present work, it was revealed that TIMP1 overexpression in BV2 cells markedly suppressed the phosphorylation levels of p38, JNK and ERK, validating the role of TIMP1 in repressing MAPK pathway in microglia.

## Conclusion

This study identified an inflammation-related biomarker in EBI of IS, TIMP1. TIMP1 regulates the transformation of microglia from pro-inflammatory M1 phenotype to anti-inflammatory M2 phenotype through MAPK pathway to reduce the neuroinflammation. Up-regulating TIMP1 may be a novel strategy for ameliorate EBI after IS.

## Electronic supplementary material

Below is the link to the electronic supplementary material.

Supplemental Fig.1. Expression of 15 core genes in the external dataset GSE107983 (A), GSE190171 (B), and GSE30655 (C) in IS and control groups

## Data Availability

The data used to support the findings of this study are available from the corresponding author upon request.
