# Supplementary figures and images for: TIMP1 promotes microglia M2 polarization through MAPK pathway to ameliorate early brain injury after ischemia
Source: Hereditas. 2025 Jul 2;162:119. doi: 10.1186/s41065-025-00491-8 (PMC12217830; doi:10.1186/s41065-025-00491-8)

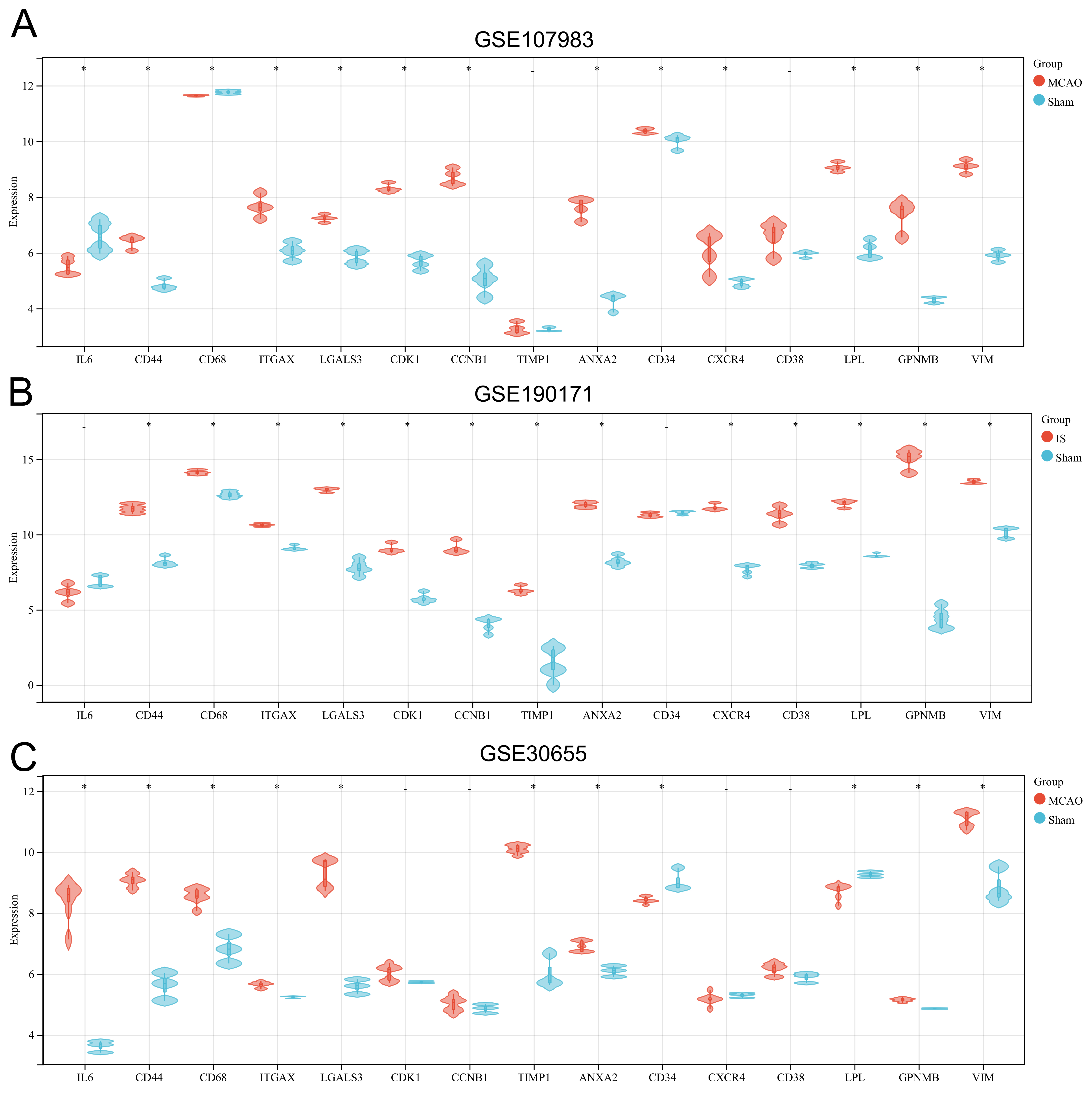

Supplement: Supplementary file 1 — Supplemental Fig.1. Expression of 15 core genes in the external dataset GSE107983 (A), GSE190171 (B), and GSE30655 (C) in IS and control groups [file 41065_2025_491_MOESM1_ESM.jpg]
